# A Suggested New Flag for Hospitals

**Published:** 1914-12-05

**Authors:** J. B. Cornish

**Affiliations:** Hon. Sec., West Cornwall Dispensary and Infirmary. Penzance


### A Suggested New Flag for Hospitals.

*To the Editor of* The Hospital.

Sir,—After five weeks I have seen your notes on the question of hospital flags. But these do not settle the question. We want a distinguishing badge or sign for use either on flags, reports, collecting-boxes, or for any other purpose, which shall clearly be understood to signify a civilian hospital. The Union Jack is of no use for these purposes, neither as a flag or a badge does it specially refer to hospitals.

As the Red Cross is now forbidden I suggest that we use a white cross of the same pattern on a red ground implying a hospital not taken over by the military authorities, having only to reverse the colours if it should be. This will make a very attractive and effective symbol, usable for every purpose required.

It is not used for any other meaning, being more different from the Danish flag than the Geneva Cross is from St. George's.

There is no authority which can give or refuse permission to use it. It is merely custom, and unless something better is suggested we propose to adopt it, and invite other hospitals to do the same.—Yours truly,

J. B. Cornish, Hon. Sec.,
West Cornwall Dispensary and Infirmary.

Penzance, November 26, 1914.

